# Bacterial and archeal community composition in hot springs from Indo-Burma region, North-east India

**DOI:** 10.1186/s13568-016-0284-y

**Published:** 2016-11-10

**Authors:** Amrita Kumari Panda, Satpal Singh Bisht, Surajit De Mandal, Nachimuthu Senthil Kumar

**Affiliations:** 1 Department of Zoology, Kumaun University, Nainital, Uttarakhand 263002 India; 2 Department of Biotechnology, Mizoram University, Aizawl, Mizoram 796004 India

**Keywords:** Hot spring, Bacterial diversity, Archaeal diversity, Community composition, Illumina sequencing

## Abstract

Bacterial and archaeal diversity of two alkaline Indian hot springs, Jakrem (Meghalaya) and Yumthang (Sikkim), were studied. Thirteen major bacterial phyla were identified of which *Firmicutes, Chloroflexi and Thermi* were dominant in Jakrem and *Proteobacteria* in Yumthang. The dominant genera were *Clostridium, Chloroflexus* and *Meiothermus* at Jakrem (water temperature 46 °C, pH 9) and *Thiobacillus*, *Sulfuritalea* at Yumthang (water temperature 39 °C, pH 8) hot springs. The four *Euryarchaeota* taxa that were observed in both the hot springs were *Methanoculleus, Methanosaeta, Methanosarcina and Methanocorposculum*. *Elstera litoralis, Thiovirga* sp., *Turneriella* sp. were observed for the first time in association with hot springs along with *Tepidibacter* sp., *Ignavibacterium* sp., *Teribacillus* sp. and *Dechloromonas* sp. Individual bacterial phyla were found to be specifically correlated with certain physico-chemical factors such as temperature, dissolved SiO_2_, elemental S, total sulphide, calcium concentrations in hot spring water. Bacterial reads involved in sulfur cycle were identified in both16S rRNA gene library and sulfur metabolism may play key physiological functions in this hot spring. Members within *Desulfobacterales* and *Thermodesulfovibrionaceae* were identified and hypothesized their role in regulating sulfur cycle. The presence of many taxonomically unsolved sequences in the 16S rRNA gene tag datasets from these hot springs could be a sign of novel microbe richness in these less known hot water bodies of Northeastern India.

**Electronic supplementary material:**

The online version of this article (doi:10.1186/s13568-016-0284-y) contains supplementary material, which is available to authorized users.

## Introduction

The Himalayas represent a unique area of geothermal system associated with continent- continent colliding zone and the Himalayan geothermal belt (HGB) extends from the north-western part to the north-eastern part of India over a length of 1500 sq km (Chauhan 2015). Geological survey of India has identified 340 hot-water springs in India and classified them on the basis of their geo-tectonic setup (Craig et al. 2013; Ghelani et al. 2015). Thermal springs located in Sikkim and Meghalaya are an integral part of HGB which is located within the Indo-Burma range and hot springs in HGB have alkaline pH and unique geochemistry i.e. elevated Na, Ca and SiO_2_ (Siangbood and Ramanujam 2011; Rakshak et al. 2013). Compared to many studies on hot springs at lower elevations such as Yellowstone National Park (Kan et al. 2011), Kamchatka in Russia (Reigstad et al. 2010), Iceland (Mirete et al. 2011) Indonesia (Aditiawati et al. 2009), Tunisia (Sayeh et al. 2010) and north-eastern Australia (Weidler et al. 2007), very little is known about the microbial diversity of high elevation Himalayan hot springs. Hot springs present in high elevation HGB are less explored in terms of biotic components (Ghosh et al. 2003). Northeast Himalayan geothermal sub-province harbors large number of thermal springs and is an important geothermal energy source in India (Razdan et al. 2008). Only a few studies have been performed on the microbial ecology of hot springs from North-eastern India (Rakshak et al. 2013; Sherpa et al. 2013) and still it is assumed that comprehensive understanding on the microbial community structure in these hot springs are less known.

Thermophilic microbial diversity is reported from many alkaline hot water springs previously (Pagaling et al. 2012; Coman et al. 2013) and it is assumed that it influenced the evolution of life on earth (Doolittle 1999). Metagenomics studies from extreme environments led to the discovery of biocatalysts, secondary metabolites and bioactive compounds (Wong 2010; Barone et al. 2014). For example sulphur-cycling genes from sulphidic deep sea hydrothermal vent communities (Cao et al. 2014), H_2_ oxidation genes from H_2_-rich serpentinite hydrothermal vent communities (Brazelton et al. 2011), lipid oxidation genes in DSHV communities (He et al. 2013) and genes for ammonia- oxidation (amoA) in the Guaymas Basin (Baker et al. 2012) were identified by community metagenome analysis.

Hot springs harbor rich bacterial diversity that could be the source of commercially important products specially enzymes, sugars, compatible solutes and antibiotics (Satyanarayana et al. 2005). Bacterial diversity analysis of such extreme environments by culture independent approaches has grown in significance because of their diverse, unusual chemistry and the opportunity they provide to identify rare compounds and genes (Kuddus and Ramtekke 2012). Hot springs of Indian subcontinent offer striking and demanding platform for researchers from the globe due to the existence of unknown and untapped microbial communities. Most of the hot springs present in Northeast of India are present in unexplored environments and their diversity studies could be of great interest to facilitate various industrial, agricultural and medicinal applications and offer potential solutions to environmental concerns including the demand for bio-fuels (Urbieta et al. 2015).

The objective of this research was to study the microbial community composition and diversity in hot springs of the HGB (Yumthang and Jakrem) located in Northeast India and to understand the influence of the hot spring physico-chemical properties on the microbial diversity. These analyses were based on the hypothesis that the alkaline hot springs of HGB will host important microbial species for bio-prospecting and that specific ecological parameters might favor the species diversity and richness.

## Materials and methods

### Sampling

**Fig. 1:**
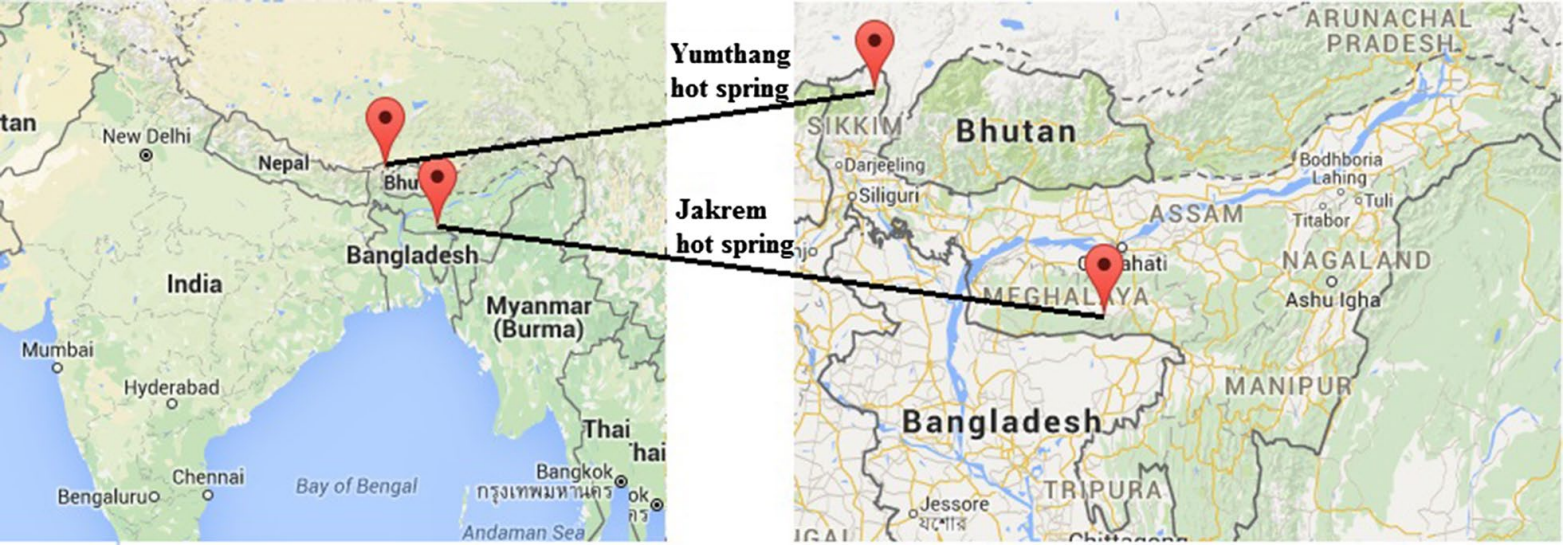
Geographical location of the sampling sites

Microbial mat along with water and sediment was collected from Jakrem, Meghalaya (temp. 46 °C; elevation of 1450 m from MSL) and Yumthang, Sikkim (temp. 39 °C; elevation of 3564 m from MSL) hot springs of Northeast India. The geographical location of the sampling sites is shown in Fig. 1. The sample was collected from random sites using a hand trowel and pooled into sterile tubes, frozen in dry ice and transported to the laboratory for further analysis. The sediment/mat color, water temperature, pH and dissolved oxygen were recorded. XRD was performed to identify the mineralogy of collected solids (Huang et al. 2011). 500–1000 ml of the sample was filtered (0.22 µm) and split into several aliquots for analysis of various anion, cation and trace elements (sodium, calcium, potassium, magnesium, iron, arsenic, phosphorous, chloride, sulfur, nitrate, aluminium, silicon, dissolved silica and total sulphide) by Inductively Coupled Plasma Optical Emission Spectroscopy3r (ICP OES-7300, Perkin Elmer, USA).

### Community DNA extraction

Total DNA was extracted from 2 to 5 g of collected sample using Fast DNA™ Spin kit for soil (MP Biomedicals, USA). The DNA concentration was quantified using a microplate reader (SpectraMax 2E, Molecular Devices, USA). Agarose gel electrophoresis of the community DNA was carried out to check the quality of the DNA, stained with ethidium bromide and visualized under gel documentation system (G-Box, SynGene, USA).

### Illumina sequencing

The V4 region of the 16S rRNA gene was amplified using 515F/806R (5′ GTGCCAGCMGCCGCGGTAA 3′; 5′ GGACTACHVGGGTWTCTAAT 3′) primers (Caporaso et al. 2012; Moonsamy et al. 2013). Cycling conditions for the PCR reaction were 98 °C for 30 s, followed by 30 cycles of 98 °C for 10 s and 72 °C for 30 s, with a 5 s elongation step at 72 °C followed by 4 °C hold. The paired-end sequencing (2 × 251 base pairs) was performed on an Illumina Mi-Seq platform at Scigenome India Pvt Ltd, Cochin, India.

### Phylogenetic and statistical analysis

QIIME data analysis package was used for 16S rRNA data analysis. Quality check on raw sequences was performed, Chimeras were removed using UCHIME, pre-processed consensus V4 sequences were grouped into operational taxonomic units (OTUs) using the clustering program UCLUST at a similarity threshold of 0.97 (Edgar 2010). The representative sequence was finally aligned against Greengenes core set of sequences using PyNAST program and representative sequence for each OTU was classified using RDP classifier and Greengenes database. Sequences which are not classified were classified as unknown. The Shannon diversity indices were calculated and it represents OTU abundance, richness and evenness. The original sequencing output files of Jakrem and Yumthang hot spring have been deposited in the Sequence Read Archive (SRA) service of the National Centre for Biotechnology Information (NCBI) database under the accession numbers *SRS932137* and *SRS932073*, respectively.

Canonical correlation analysis was performed to determine the correlation between microbial diversity and geochemical factors using PAST: Paleontological statistics software (Hammer et al. 2001). Pearson correlation between hot spring physico-chemical parameters and bacterial phyla were calculated using PASW statistics 18 (SPSS Inc, Chicago, USA).

### Analysis of metabolic potential

The bioinformatics pipeline PICRUSt (Phylogenetic Investigation of Communities by Reconstruction of Unobserved States) (Langille et al. 2013) was used to address the functional potential of the microbes present in the hot springs. The closed-reference OTU picking protocol using QIIME 1.9.1 (Caporaso et al. 2012) was used and sequences were searched against the Greengenes database, version 13_05, taxonomically assigned using uclust with default parameters (Edgar 2010). The OTU table was created and analyzed by PICRUSt pipeline. The PICRUSt pipeline scans KEGG functional database and uses the OTU table of assigned taxa and their relative distribution to generate the relative abundance of functional categories. Data produced by the PICRUSt pipeline was statistically evaluated with the STAMP bioinformatics package (Parks and Beiko 2010).

## Results

### Geochemical analysis

Microbial mats in Jakrem were green in color whereas white microbial mats and grey macroscopic filaments observed at Yumthang. The temperature, pH, dissolved elemental composition and mineralogical data of the hot springs are described in Tables 1 and 2. The aqueous concentrations of cations such as sodium, calcium and potassium were highest in Jakrem hot spring, where as total sulphide concentration was high in Yumthang hot spring. XRD analysis shows that quartz is predominant in both hot spring sediments, whereas other minerals included are tridymite, wollastonite and kyanite (Table 2).

**Table 1:** Dissolved elemental analysis of the samples

Research site	pH	Parameters															
		Temp. (°C)	DO (mg/l)	Na (mg/l)	Ca (mg/l)	K (mg/l)	Mg (mg/l)	Fe (mg/l)	As (mg/l)	P (mg/l)	Cl (mg/l)	S (mg/l)	NO_3_ (mg/l)	Al (mg/l)	Si (mg/l)	Dissolved SiO_2_ (mg/l)	Total sulphide (mg/l)
Jakrem	9–10	46	3.0	69.25	3.48	0.875	0.239	0.023	<0.01	0.054	13.20	12.69	0.012	0.093	16.75	12.82	<0.01
Yumthang	8–9	39	4.0	49.25	1.56	0.66	<0.01	0.015	<0.01	0.104	21.83	6.62	<0.01	<0.01	4.60	7.55	0.16

**Table 2:** Rock mineral analysis by Xpert High score software

Research site	High score minerals in descending order of their abundance
Jakrem	Quartz > Tridymite > Raspite > Wollastonite > Rankinite > Kyanite > Forsterite > Clinoenstatite > Tungstite SiO_2_ > SiO_2_ > PbWO_4_ > CaSiO_3_ > Ca_3_Si_2_O_7_ > Al_2_O_3_.Si O_2_ > Mg_2_SiO_4_ > Mg SiO_3_ > WO_3_
Yumthang	Quartz > Wollastonite > Cuprite > Molybdite > Tenorite > Kyanite SiO_2_ > CaSiO_3_ > Cu_2_O > MoO_3_ > CuO > Al_2_O_3_.Si O_2_

### Analysis of bacterial diversity

**Fig. 2:**
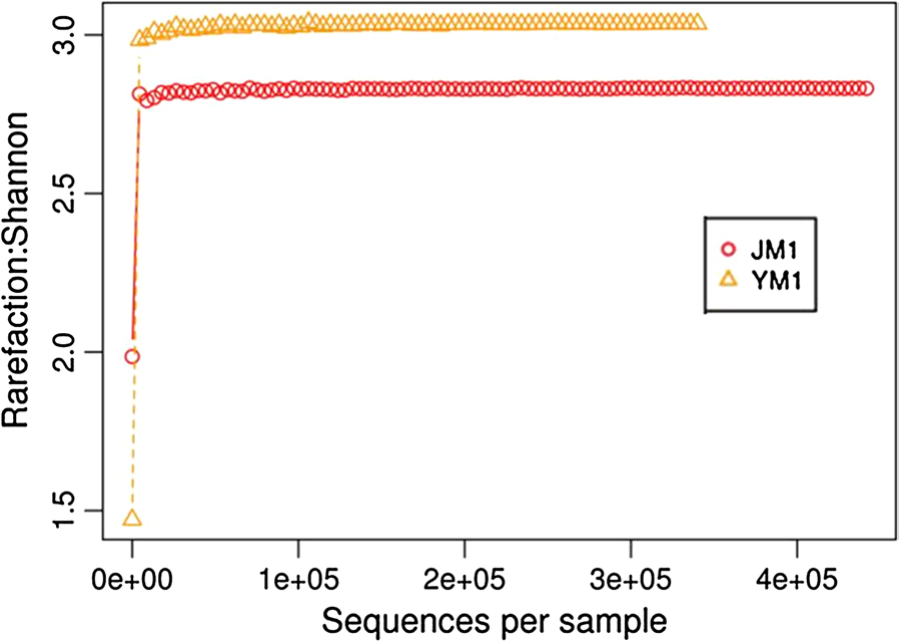
Rarefaction analysis of alpha diversity among JM1 and YM1. Shannon diversity matrix was used

Alpha diversity indices including Shannon, Chao1 and observed species metrices showed that sample YM1 is more diverse. The Shannon index was 2.10 and 1.96 for the Yumthang and Jakrem hot spring, respectively. Similarly the number of OTU and Chao index was higher in Yumthang as compared to Jakrem hot spring (Table 3). The metric calculation was performed using QIIME software. Rarefaction curve for Shannon metric indicated that the sample has reached near saturation for higher taxonomic levels (Fig. 2).

**Table 3:** Diversity indices for of hot spring microbial communities

Sample name	Shannon–Weaver index (H)	Chao 1	Number of OTU
JM1	1.963	1561	561
YM1	2.10	1891	891

### Bacterial, archaeal diversity and community composition

The bacterial and archaeal diversity was assessed by illumina sequencing of V4 hyper-variable region of bacterial and archaeal small sub-unit rRNA genes with reference to these two unexplored hot springs of North- Eastern India. Jakrem 16S rRNA gene library comprised of 682,049 reads with 342.38 Mb data and average sequence length of 251 bp (Table 4). The G + C content was 57.87% and more than 90% sequence had a Phred score >=Q30. A total of 509,150 raw sequences (255. 59 Mb data) were obtained from Yumthang mat high throughput 16S rRNA library. These sequence reads clustered into Operational Taxonomic Units (OTUs) based on their sequence similarity using Uclust program (similarity cutoff = 0.97). A total of 881, 622 preprocessed reads were clustered into 1188 OTUs. Sample libraries ranged from 540,082 (JM1) to 341,540 (YM1) sequence reads (Table 4). Approximately, 561 OTUs were obtained from Jakrem, whereas 891 OTUs found in Yumthang library. Forty OTUs from Jakrem and 80 OTUs from Yumthang high throughput 16S metagenome library didn’t cluster with any of the previously known microbial classifications.

**Table 4:** Pre-processing read statistics of illumina paired-end reads

Sample name	Total reads	Passed conserved region filter	Passed spacer	Passed read quality filter	Passed mismatch filter	Consensus reads	After singleton removal	Chimeric sequences	Pre-processed reads
JM1	682,049	627,554	625,976	625,830	577,872	577,872	540,719	637	540,082
YM1	509,150	461,665	461,019	460,943	402,969	402,969	342,830	1290	341,540

The archaeal community consisted of sequences closely related to *Euryarchaeota* and *Crenarchaeota* in both springs. Two identified order under the phyla *Euryarchaeota* were *Methanomicrobiales* and *Methanosarcinales*. Members under *Crenarchaeota* belonged to Marine Benthic Group (MBGA). Archaeal diversity did not show much variation between the two study sites.

**Fig. 3:**
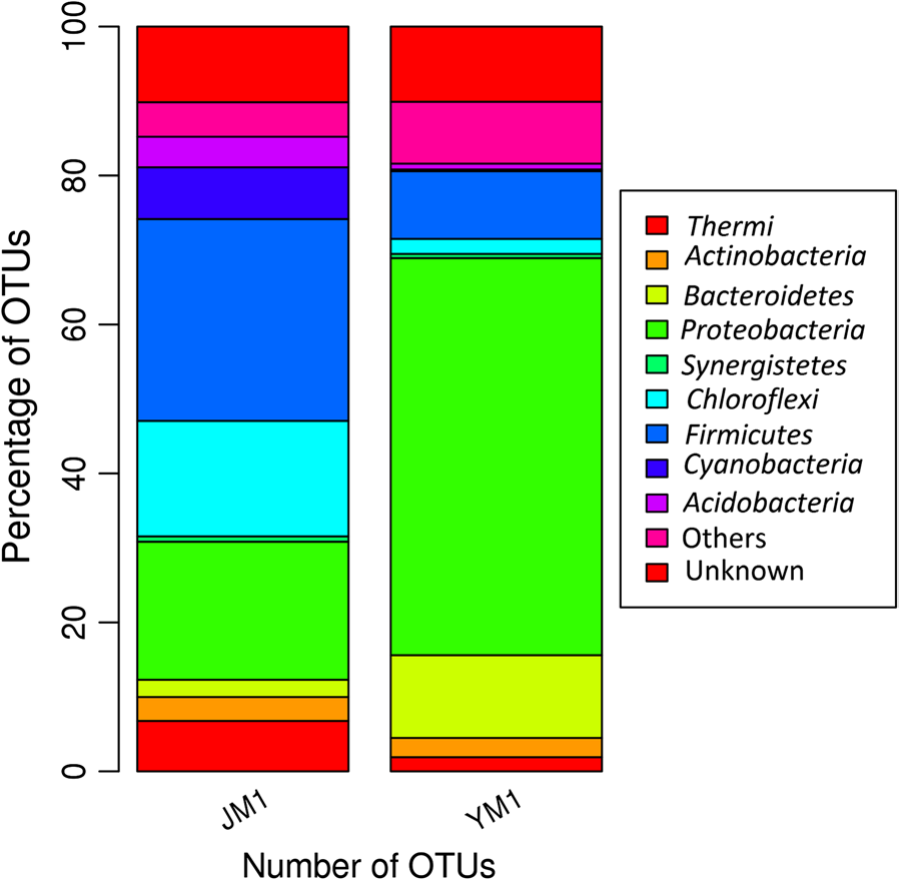
Taxonomic classification of OTUs at phylum level (JM1—Jakrem samples, YM1—Yumthang samples)

**Fig. 4:**
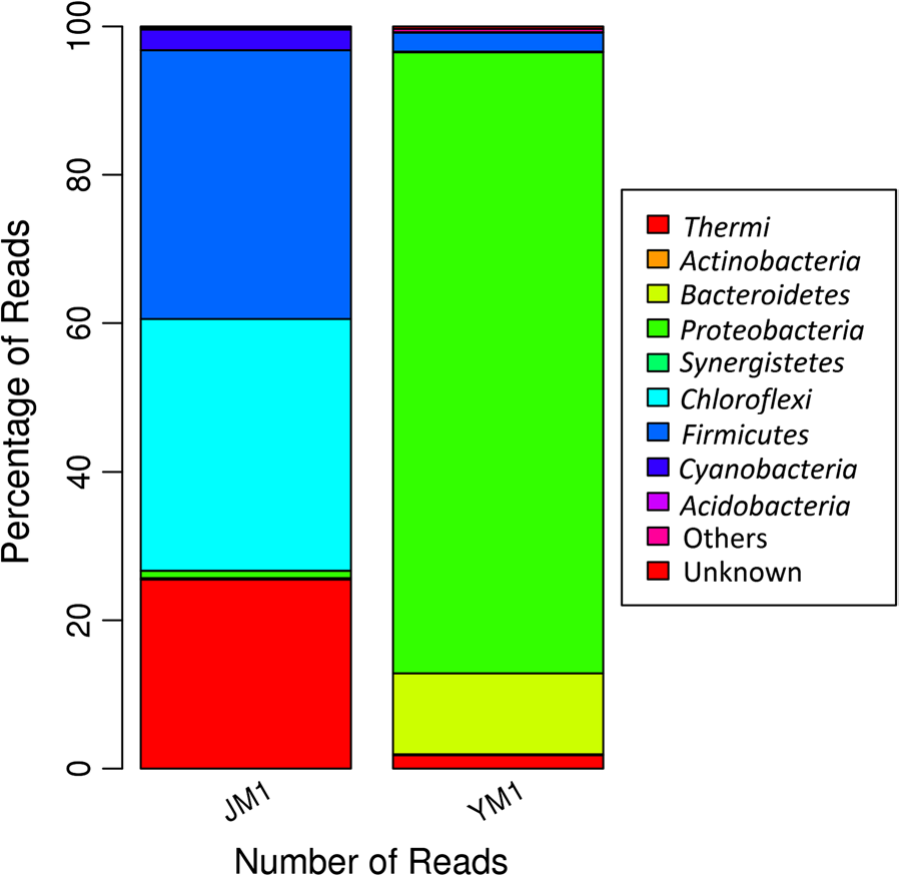
Taxonomic classification of reads at phylum level (JM1—Jakrem samples, YM1—Yumthang samples)

The phylum level bacterial diversity identified in the high throughput 16S rRNA libraries from JM1 (Jakrem) and YM1 (Yumthang) hot springs is presented in Figs. 3 and 4. A total of 19 distinct phyla in the Yumthang 16S rRNA library dominated by *Proteobacteria* (83.68%), *Bacteroidetes* (10.93%) and *Thermi* (1.78%) whereas Jakrem 16S rRNA library accounted for 36.08% of *Firmicutes*, 34.18% of *Chloroflexi* and 25.44% of *Thermi* (Fig. 4).

Thermophilic OTUs belonging to the *alpha*-*, beta*-*, delta*- and *gammaproteobacteria* were detected in both the samples but with variable abundance. The four classes of *Proteobacteria* are represented by the families *Rhodobacteraceae, Caulobacteraceae, Oxalobacteraceae, Comamonadaceae, Thiotrichaceae, Moraxellaceae, Halomonadaceae, Halothiobacillaceae, Desulfomicrobiaceae and Desulfobacteraceae*. The most abundant taxonomic groups among *Proteobacteria* are *Betaproteobacteria* (82%) represented by dominated OTU 617 classified further under the order *Rhodocyclales* with other *Proteobacterial* sequences affiliated with *Alphaproteobacteria* (0.79%), *Gammaproteobacteria* (0.36%) and *Deltaproteobacteria* (0.33%) in the hot spring microbial mat of Yumthang. The other dominant OTUs within *Betaproteobacteria* were OTU 128, 1320 and 940 were classified under the order *Burkholderiales, Thiobacterales* and *Hydrogenophilales* respectively (Additional file 1: Table S1). Previous studies show the predominance *Proteobacteria*, particularly of *Betaproteobacteria* in a circumneutral hot spring from the Uzon Caldera, Kamchatka, Russia (Wemheuer et al. 2013; Chan et al. 2015) and other acidic thermal springs (Wilson et al. 2008). The information on distribution of genera is listed in Additional file 1: Table S2.

### PICRUSt analysis

PICRUSt uses the OTU table of assigned taxa and their relative distribution to generate the relative abundance of functional categories based on sequenced genomes. On the basis of different KEGG functional gene ontology, the functions are arranged into five functional modules: metabolism, genetic information processing, environmental information processing, cellular processes, organismal systems. The fact that no differences were observed among both the samples via. PICRUSt analysis (Additional file 1: Figure S1) could be attributed to a low quantity and quality of annotated genomes that are related to the species observed in the hot spring samples. The increase of prevalence of genes encoding carbon fixation through photosynthesis in Jakrem can be explained by the high diversity of phototrophs (Additional file 1: Figure S1). Methane and Sulfur metabolism gene modules were also identified by PICRUSt pipeline accounting for the role of methane and sulfur in the regulation of geochemical cycle. The most abundant gene categories were purine, pyrimidine, arginine, proline, amino sugar and nucleotide sugar metabolites which reflects the basic requirements of microbial life.

### Linking microbial community structure and hot spring geochemistry

**Fig. 5:**
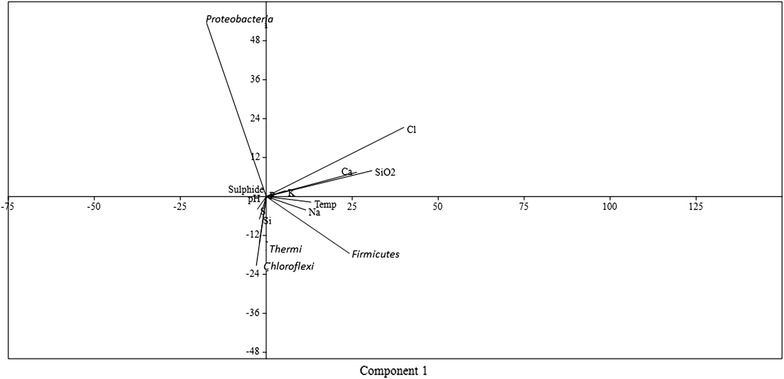
Correlative relationship between dominant microbial phyla and geochemical factors (Temperature, pH, Na, Ca, Cl, dissolved SiO_2_, K, elemental S, total sulphide and Si)

Principal component analysis was used to analyze the major geochemical factors responsible for shaping the microbial community structure in the microbial mat of both the hot springs. Simultaneously, the microbial community and geochemical parameters from Manikaran hot spring, India (north western Himalayas) (Bhatia et al. 2015; Chandrasekharam et al. 2005) and Sungai Klah (SK) alkaline hot spring, Malaysia (Chan et al. 2015) were taken into account to overcome the problem of small sample size in the present study for two component analysis. PCA method showed that the community composition was significantly (p < 0.05) linked to temperature, dissolved SiO_2_, elemental S, total sulphide, calcium etc. (Fig. 5). *Thermi* and *Chloroflexi* were negatively correlated with phosphorous (p < 0.01). The correlation analysis showed that few dominant bacterial phyla were positively correlated with particular geochemical factor such as the *Firmicutes* with temperature, Ca, Cl, dissolved SiO_2_; *Thermi* and *Chloroflexi* with pH, Si and elemental S; *Proteobacteria* specifically correlates with total sulphide (Additional file 1: Table S3; Fig. 5).

## Discussion

The taxonomic and metabolic compositions of microbial communities are both shaped and constrained by the characteristics of their local environment (Alsop et al. 2014). There are recent published studies focusing on few environmental parameters i.e. temperature and pH to determine the evolution of thermophilic microbial communities (Cowan et al. 2015). The present study is an attempt to investigate the factors determining the composition of thermophilic microbial communities by including multiple geochemical parameters. The present investigation characterized the archaeal and bacterial communities of two alkaline springs with similar temperature and pH, but different geochemical parameters. The archaeal and bacterial diversity varied across both the hot springs.

*Tepidimonas taiwanensis* and *Tepidimonas fonticaldi* are both thermophilic bacteria isolated from a hot spring of southern Thailand (Chen et al. 2006) and Antun hot spring from Taiwan (Chen et al. 2013). This study also identified few *Tepidimonas* OTUs in Jakrem 16S rRNA library. It is the third description worldwide in association with hot springs. Members of the genus *Tepidimonas* were shown to produce strong alkaline protease activity (Chen et al. 2006). One hundred thirty four V4 16S rDNA reads from the Yumthang library share more than 90% identity with members of *Dechloromonas* group those are poorly described from other thermal environments. There are only three type species reported from genus *Dechloromonas* i.e. *Dechloromonas agitata* (Achenbach et al. 2001); *Dechloromonas denitrificans* (Horn et al. 2005); *Dechloromonas hortensis* (Wolterink et al. 2005) till date. The microbes isolated from Gedongsongo hot spring (pH 6.0–7.0) Indonesia were closely related to *Dechloromonas* genera (Aminin et al. 2008). *Dechloromonas* represents a unique genus with a broad range of novel metabolic capabilities and bioremediative applicability. There are reports that *Dechloromonas* strains RCB and JJ can completely mineralize various mono-aromatic compounds including benzene to CO_2_ in the absence of O_2_ with nitrate as the electron acceptor (Coates et al. 2001). PICRUSt predicted the presence of certain xenobiotic metabolizing genes during the present investigations (Additional file 1: Figure S1).

Partial 16SrDNA sequences from Yumthang and Jakrem hot springs share more than 96% identity with the members of *Thiobacillus sajanensis* strain 4G. This is a new obligate autotrophic sulfur-oxidizing bacterium isolated from Khoito-Gol hydrogen-sulfide springs in Buryatia (Dul’tseva et al. 2006). The bacteria belonging to *Thiobacillus* group are mesophilic to moderately thermophilic and were found in thermal environments of Africa (Khavarpour et al. 2011), Italy (Pentecost et al. 2004), New Zealand (Jones and Renaut 2007) and Romania (Coman et al. 2013). V4 16S rDNA reads from Yumthang share identity with *Sulfuritalea hydrogenivorans*, a novel sulfur-oxidizing *betaproteobacterium*. *Delta* p*roteobacterial* 16S rDNA sequences observed from Yumthang library are closely related to *Desulfomicrobium baculatum*, a gram-negative, motile, sulfate-reducing bacterium isolated from water-saturated manganese carbonate ore (Copeland et al. 2009).

The most dominant OTU within *Chloroflexi* was OTU 1166 classified under the genus *Chloroflexus* present in Jakrem were closely related to *Chloroflexus aurantiacus* strain J-10-fl which is also reported from microbial mats of various neutral to alkaline hot springs of the world viz. Romania (Coman et al. 2013); China (Lau and Pointing 2009; Lau et al. 2009); Thailand (Kanokratana et al. 2004; Portillo et al. 2009); Japan (Sekiguchi et al. 2003); USA (Costa et al. 2009); France (Gregoire et al. 2011a, b) or Russia (Bryanskaya et al. 2006). The *Chloroflexus* is a filamentous anoxygenic phototrophic bacterium. At taxonomic level, four *Chloroflexi* OTUs from Yumthang showed 87% similarity with *Thermomarinilinea lacunofontalis* strain SW7 isolated from the main hydrothermal vent of the Taketomi submarine hot spring field located on the southern part of Yaeyama Archipelago, Japan. These OTUs were encountered in similar hot springs from China (Lau and Pointing 2009; Lau et al. 2009), Thailand (Kanokratana et al. 2004; Portillo et al. 2009), Japan (Sekiguchi et al. 2003), USA (Costa et al. 2009), France (Gregoire et al. 2011a, b), Russia (Bryanskaya et al. 2006) and Romania (Coman et al. 2013).

Photosynthetic *Cyanobacterial* reads detected in Jakrem hot spring are closely related to filamentous *Cyanobacterial Arthronema* and *Leptolyngbya* genera. Some *Oscillatoriales Cyanobacteria* (especially *Leptolyngbya* sp.) were observed to dominate hot springs with temperature of about 55 °C (Roeselers et al. 2007; McGregor and Rasmussen 2008; Sompong et al. 2008). Their filamentous structures and polysaccharide matrices probably represent the backbone of the microbial mat (Van Gemerden 1993). The metabolic abilities of filamentous members of the *Oscillatoria* order can shed light on the role played by closely related clones encountered in the Jakrem spring mats. *Oscillatoria* species are known to dwell in anaerobic, sulfide-containing habitats (Castenholz 1989). At night they can grow by fermenting glycogen and other compounds produced during day time photosynthesis. Some species are also capable of growth in the dark via sulfur respiration (Richardson and Castenholz 1987; Teske and Stahl 2002). Elemental sulfur is produced as an intermediate of anoxygenic photosynthesis and is abundant in the Jakrem spring. Therefore, the anoxic conditions (3.0 mg/l dissolved oxygen along with high elemental sulfur concentrations) in the Jakrem spring represent an ideal habitat for members of the order *Oscillatoriales*.

The occurrence “*Candidatus* Xiphinematobacter,” in Jakrem 16S rRNA library is of interest as only few species reported till date. ‘*Candidatus* Xiphinematobacter’ gen. nov., along with three new candidate species, ‘*Candidatus* Xiphinematobacter americani’ sp. nov., ‘*Candidatus* Xiphinematobacter rivesi’ sp. nov. and *‘Candidatus* Xiphinematobacter brevicolli’ sp. nov., were reported (Vandekerckhove et al. 2000). The non-*Proteobacterial* obligately methanotrophic bacterium Kam1 belonging to the *Verrucomicrobia* was recovered from an acidic hot spring in Kamchatka, Russia and is more thermoacidophilic than any other known methanotroph with optimal growth at 55 °C and pH 3.5 (Islam et al. 2008). The majority of the *Firmicutes* sequences in Jakrem 16S rRNA library were affiliated with the genus *Clostridium*. The most dominant OTU among the *Firmicutes* phyla is OTU 1235 (read = 154,229), having close sequence similarity with the *Clostridium* sp. TB10. Approximately, 25% of the total bacterial clone sequences in Jakrem library were closely related to *Deinococcus*—*Meiothermus*. The *Bacteroidetes* sequences observed in the Yumthang library and *Flavobacterium* represented the predominant genus of this phylum.

Other bacterial reads such as *Elstera litoralis, Thiovirga* sp., *Turneriella* sp. were observed for the first time in association with the hot spring. The presence of sequence reads from bacterial taxa *Tepidibacter* sp., *Ignavibacterium* sp., *Teribacillus* sp., *Dechloromonas* sp., could be the representatives of novel species within these genera. The genus *Tepidibacter* (*Firmicutes*) was proposed (Slobodkin et al. 2003) with three reported type species i.e. *Tepidibacter thalassicus, Tepidibacter formicigenes and Tepidibacter mesophilus* (Tan et al. 2012). Thus, the OTUs from Jakrem hot spring representing *Tepidibacter* (*Firmicutes*) possibly are the novel species with in this genus. The bacterium *Ignavibacterium album* was reported to be the only members of the bacterial phylum *Chlorobi* (Iino et al. 2010; Liu et al. 2012). *Ignavibacterium* was the only member of the phylum *Chlorobi* detected in these two hot springs. The presence of number of taxonomically unsolved sequence reads in both the hot spring high throughput 16S rRNA library is a sign of many novel microbes indigenous to these selected hot water springs. The findings of the molecular survey of these two so far not investigated sites showed that these hot springs are repository of unique bacterial and archaeal species in the biodiversity rich regions of the world.

Low diversity of archaea was found with genus-level OTUs corresponding to *Methanocorposculum, Methanosaeta and Methanosarcina* in both the springs and with an addition of *Methanoculleus* in Yumthang hot spring alone. Methanogenic microbes can use H_2_, acetate, formate, methanol, carbon monoxide and various methylamines as energy sources (Balch 1979). Methane could be playing a major role in geochemical cycling at Yumthang hot spring which is indicated by the presence of methanogenic archaeal sequence reads and methane metabolism genes in in silico analysis by PICRUSt (Additional file 1: Figure S1) in this environment. Newell et al. (2008) measured gas concentrations at various springs along the southern margin of the Tibetan plateau and observed variable CH_4_ concentrations from 110 to 500 ppm. The presence of methanogenic reads in Yumthang spring indicates methane may be derived from deeply buried carbon-bearing rocks or it could be produced in the near surface by organic matter fermentation (Whiticar 1986).

Thermophilic strains of *Methanosarcina* sp. have been reported with growth temperature of 55 °C (Zinder and Mah 1979) and *Methanosaeta thermophila* (Nozhevnikova and Yagodina 1983) from a hot spring (55 °C) of Kamchatka, Russia. However, the occurrence of *Methanosarcina* and *Methanosaeta* sequence reads in Yumthang hot spring 16S rRNA illumina library (water temperature 39 °C) suggests that there are members of both of these genera that grow at lower temperatures (similar to and below that found in Yumthang). There are reports on occurrence of methanogens including *Methanomicrobiales* and *Methanosarcinales* and relatives of *Methanomassiliicoccus luminyensis* from hot springs of Armenia (Hedlund et al. 2013). Our study revealed the dominance of *Euryarchaeota* over *Crenarchaeota* in these hot springs.

*Thermi* and *Chloroflexi* were negatively correlated with phosphorous (p < 0.01). The correlation analysis showed that few dominant bacterial phyla were positively correlated with particular geochemical factor such as the *Firmicutes* with temperature, Ca, Cl, dissolved SiO_2_; *Thermi* and *Chloroflexi* with pH, Si and elemental S; *Proteobacteria* specifically correlates with total sulphide (Additional file 1: Table S3; Fig. 5). *Desulfomicrobiaceae and Desulfobacteraceae. Thermi* and *Chloroflexi* were negatively correlated with phosphorous (p < 0.01). The correlation analysis showed that few dominant bacterial phyla were positively correlated with particular geochemical factor such as the *Firmicutes* with temperature, Ca, Cl, dissolved SiO_2_; *Thermi* and *Chloroflexi* with pH, Si and elemental S; *Proteobacteria* specifically correlates with total sulphide (Additional file 1: Table S3; Fig. 5). In the complete dataset, only few significant correlations were observed between hot spring geochemical factors and dominant phyla which may be attributed to small sample size in the present study.

**Fig. 6:**
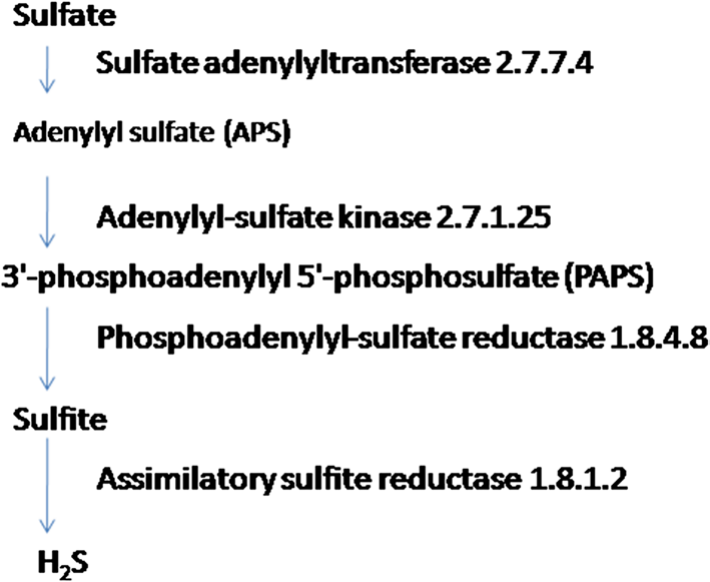
KEGG based analysis of sulfur metabolism

As the two hot springs from Jakrem and Yumthang showed small differences in temperature and pH, the difference in bacterial community may be due to differences in aqueous geochemistry. Sulfur metabolism involves sulfur oxidation and sulfur reduction both. Because reads detected in 16S rRNA gene library were closest to the sulfate reducing microbes, we conclude that both hot spring communities preferably generate the reductive form of sulfur compounds (Fig. 6). The Yumthang hot spring had low dissolved oxygen (4 mg/l) and alkaline pH and these conditions appear to favour sulfate-reducing microorganisms. For example, the reads of *Deltaproteobacterial* order *Desulfobacterales* (Widdel 1987), *Thermodesulfovibrionaceae* family from *Nitrospirae* (Haouari et al. 2008) capable of reducing sulfates to sulfides were identified. The presence of large amount of total sulphide (0.16 mg/l) (Table 1) in Yumthang may be possible due to the presence of sulfate-reducing micro organisms.

Sulfate-reducing microorganisms are important in degrading organic matter under anoxic environments. In the Jakrem community, the organisms related to sulfate reduction identified from library those having closest hits to *Desulfomicrobium apsheronum* (Rozanova et al. 1988), *Desulfomicrobium thermophilum* (Thevenieau et al. 2007) and *Desulfomicrobium* sp. B1 (Chen et al. 2008). These sulfate-reducing microorganisms play an important role in energy production as well as the maintenance of the microbial community (Elshahed et al. 2003; Douglas and Douglas 2001). They depend on sulfate and elemental sulfur as the terminal electron acceptor during anaerobic metabolism. Reduction of sulfate to sulfite was activated by the formation of adenosine-5′-phosphosulfate (APS) and 3′-phosphoadenosine-5′-phosphosulfate (PAPS) which was further reduced to sulfite and hydrogen sulfide using the enzyme of phosphoadenosine phosphosulfate reductase and sulfite reductase. The abundance of δ-*Proteobacteria* and purple sulfur γ-*Proteobacteria* (80–83%) in the microbial mat bacterial diversity of the studied North east Indian hot spring samples was consistent with previous observations in mesophilic sulfide-rich springs (Elshahed et al. 2003).

## Conclusions

This culture-independent study has provided an important insight into the potentially novel microbial diversity and community composition of two alkaline hot springs of Himalayan Geothermal Belt. Jakrem hot spring (39 °C) was abundant with the bacterial genera *Clostridium, Chloroflexus* and *Meiothermus* whereas *Thiobacillus*, *Sulfuritalea* was abundant in Yumthang (45 °C) hot springs. Bacterial phyla were found to be specifically correlated with physico-chemical factors of the hot spring water such as the *Firmicutes* with temperature, Ca, Cl, dissolved SiO_2_; *Thermi* and *Chloroflexi* with pH, Si and elemental S; *Proteobacteria* specifically correlates with total sulphide. Several bacterial genera with known industrial importance were identified from the hot spring metagenomes. The presence of high sulphide concentration as well as sulfate-reducing micro organisms in Yumthang indicates an active sulphur cycle in this hot spring. Many sequence reads not closely similar to any of the known species identified in the present study indicates the possibility of novel microbes in these habitats. Further studies with cultivation followed by physiological analysis of these important microbes would be required to determine their precise functional roles within these communities.

## Additional file

**Additional file 1: Table S1.** Top fifteen OTU’s based on total read count number among the hot spring samples.** Table S2.** The distribution of genera in two samples.** Table S3.** Correlation matrix showing r values for Pearson’s correlation. **Figure S1.** Relative abundance of genes in the two hot spring samples (orange color: Yumthang hot spring, blue color: Jakrem hot spring) for selected functional KEGG pathways inferred from 16S rRNA gene data using PICRUSt.
