# Supplementary material for: Bacterial and archeal community composition in hot springs from Indo-Burma region, North-east India
Source: AMB Express. 2016 Nov 10;6:111. doi: 10.1186/s13568-016-0284-y (PMC5104702; doi:10.1186/s13568-016-0284-y)
Supplement: Supplementary file 1 — Additional file 1: Table S1. Top fifteen OTU’s based on total read count number among the hot spring samples. Table S2. The distribution of genera in two samples. Table S3. Correlation matrix showing r values for Pearson’s correlation. Figure S1. Relative abundance of genes in the two hot spring samples (orange color: Yumthang hot spring, blue color: Jakrem hot spring) for selected functional KEGG pathways inferred from 16S rRNA gene data using PICRUSt. [file 13568_2016_284_MOESM1_ESM.docx]

**Article title**: Bacterial community composition in two hot springs from Indo-Burma region, North-east India

**Journal name**: AMB Express

**Author names**: Amrita Kumari Panda^1*^, Satpal Singh Bisht^1^, Surajit De Mandal^2^, Nachimuthu Senthil Kumar^2^

**Affiliation:**

^1^Department of Zoology, Kumaun University, Nainital-263002, Uttarakhand, India.

^2^Department of Biotechnology, Mizoram University, Aizawl-796004, Mizoram, India.

**e-mail address of the corresponding author**: [itu.linu@gmail.com](mailto:itu.linu@gmail.com)

**Table S1 Top fifteen OTU’s based on total read count number among the hot spring samples.**

| **OTU Table Id** | **Read count** | **JM1** | **YM1** | **Phylum** | **Class** | **Order** | **Family** | **Genus** |
| --- | --- | --- | --- | --- | --- | --- | --- | --- |
| denovo 617 | 216063 | 714 | 215349 | *Proteobacteria* | *Betaproteobacteria* | *Rhodocyclales* | *Rhodocyclaceae* | *---* |
| denovo1166 | 169317 | 169295 | 22 | *Chloroflexi* | *Chloroflexi* | *Chloroflexales* | *Chloroflexaceae* | *Chloroflexus* |
| denovo1235 | 154245 | 154229 | 16 | *Firmicutes* | *Clostridia* | *Clostridiales* | *Clostridiaceae* | *Clostridium* |
| denovo857 | 128558 | 124698 | 3860 | *Thermi* | *Deinococci* | *Thermales* | *Thermaceae* | *Meiothermus* |
| denovo988 | 23281 | 55 | 23226 | *Bacteroidetes* | *Bacteroidia* | *Bacteroidales* | *GZKB119* | *---* |
| denovo51 | 16798 | 16798 | 0 | *Firmicutes* | *Clostridia* | *Clostridiales* | *Clostridiaceae* | *Clostridium* |
| denovo890 | 13842 | 13842 | 0 | *Cyanobacteria* | *Synechococcophycideae* | *Pseudanabaenales* | *Pseudanabaenaceae* | *Arthronema* |
| denovo676 | 9837 | 31 | 9806 | *Bacteroidetes* | *Bacteroidia* | *Bacteroidales* | *Rikenellaceae* | *Blvii28* |
| denovo128 | 9631 | 39 | 9592 | *Proteobacteria* | *Betaproteobacteria* | *Burkholderiales* | *Oxalobacteraceae* | *---* |
| denovo364 | 7235 | 44 | 7191 | *Firmicutes* | *Clostridia* | *Clostridiales* | *---* | *---* |
| denovo810 | 6578 | 6177 | 401 | *Thermi* | *Deinococci* | *Thermales* | *Thermaceae* | *Meiothermus* |
| denovo1320 | 6196 | 28 | 6168 | *Proteobacteria* | *Betaproteobacteria* | *Thiobacterales* | *---* | *---* |
| denovo721 | 5735 | 12 | 5723 | *Proteobacteria* | *Betaproteobacteria* | *---* | *---* | *---* |
| denovo1273 | 5340 | 5340 | 0 | *Firmicutes* | *Clostridia* | *Clostridiales* | *Clostridiaceae* | *Clostridium* |
| denovo940 | 4402 | 22 | 4380 | *Proteobacteria* | *Betaproteobacteria* | *Hydrogenophilales* | *Hydrogenophilaceae* | *Thiobacillus* |

**Table S2 The distribution of genera in two samples**

| Genera | Yumthang | Jakrem | Genera | Yumthang | Jakrem | Genera | Yumthang | Jakrem |
| --- | --- | --- | --- | --- | --- | --- | --- | --- |
| *Acidaminobacter* | *+* | *_* | *Desulfomicrobium* | *+* | *+* | *Meiothermus* | + | + |
| *Acinetobacter* | *+* | *+* | *Dechloromonas* | *+* | *_* | *Methanocorpusculum* | + | + |
| *Alteromonas* | *+* | *-* | *Elstera* | *+* | *_* | *Methanoculleus* | + | _ |
| *Arthrobacter* | *+* | *+* | *Ethanoligenens* | *+* | *_* | *Methanosaeta* | + | + |
| *Arthronema* | *_* | *+* | *Exiguobacterium* | *+* | *_* | *Methanosarcina* | + | + |
| *Aminiphilus* | *+* | *_* | *Flavobacterium* | *+* | *+* | *Mycobacterium* | + | + |
| *Anaerovorax* | *+* | *_* | *Flectobacillus* | *+* | *_* | *Mycoplana* | + | + |
| *Asticcacaulis* | *+* | *_* | *Fluviicola* | *+* | *_* | *Polaromonas* | + | + |
| *Bacillus* | *_* | *+* | *Fusibacter* | *+* | *_* | *Providencia* | + | + |
| *Bdellovibrio* | *+* | *_* | *Gemmata* | *_* | *+* | *Pseudomonas* | + | + |
| *Chloroflexus* | *+* | *+* | *Halomonas* | *+* | *+* | *Psychrobacter* | + | + |
| *Clostridium* | *+* | *+* | *Ignavibacterium* | *+* | *+* | *Rhodobacter* | + | _ |
| *Candidatus Xiphinematobacter* | *_* | *+* | *Kosmotoga* | *+* | *_* | *Rhodococcus* | + | + |
| *Carnobacterium* | *+* | *_* | *Lactococcus* | *_* | *+* | *Roseococcus* | + | + |
| *Chryseobacterium* | *+* | *_* | *Leptolyngbya* | *_* | *+* | *Rubrivivax* | + | + |
| *Corynebacterium* | *+* | *_* | *Massilia* | *+* | *_* | *Ruminococcus* | _ | + |
| *Sedimentibacter* | *+* | *+* | *Thiovirga* | *+* | *-* | *Treponema* | + | - |
| *Shewanella* | *+* | *+* | *Sulfuritalea* | *+* | *+* | *Terribacillus* | + | + |
| *Sphingomonas* | *+* | *+* | *Tepidibacter* | *_* | *+* | *Thiobacillus* | + | + |
| *Streptomyces* | *+* | *+* | *Tepidimonas* | *_* | *+* | *Thiofaba* | + | - |
| *Thiothrix* | *+* | *-* | *Turneriella* | *+* | *-* |  |  |  |

**Table S3 Correlation matrix showing r values for Pearson’s correlation.**

**. Correlation is significant at the 0.01 level (2-tailed),

*. Correlation is significant at the 0.05 level (2-tailed).

| Phyla | | pH | Temp | Ca | P | Cl | S | Si | SiO2 | Sulphide | Na | K |
| --- | --- | --- | --- | --- | --- | --- | --- | --- | --- | --- | --- | --- |
| *Thermi* | Pearson Correlation | **.926** | -.310 | -.333 | -.995^**^ | -.346 | .878 | **.967^*^** | -.264 | -.318 | .409 | -.331 |
|  | Sig. (2-tailed) | .074 | .690 | .667 | .005 | .654 | .122 | .033 | .736 | .682 | .591 | .669 |
| *Firmicutes* | Pearson Correlation | -.470 | **.966^*^** | **.881** | .012 | .805 | -.569 | -.294 | .865 | -.745 | .512 | .870 |
|  | Sig. (2-tailed) | .530 | .034 | .119 | **.988** | .195 | .431 | .706 | .135 | .255 | .488 | .130 |
| *Chloroflexi* | Pearson Correlation | **.919** | -.301 | -.343 | -.997^**^ | -.366 | .865 | **.949** | -.279 | -.370 | .370 | -.342 |
|  | Sig. (2-tailed) | .081 | .699 | .657 | .003 | .634 | .135 | .051 | .721 | .630 | .630 | .658 |
| *Proteobacteria* | Pearson Correlation | -.066 | -.622 | -.451 | .492 | -.337 | .055 | -.169 | -.451 | **.975^*^** | -.395 | -.436 |
|  | Sig. (2-tailed) | .934 | .378 | .549 | .508 | .663 | **.945** | .831 | .549 | .025 | .605 | .564 |


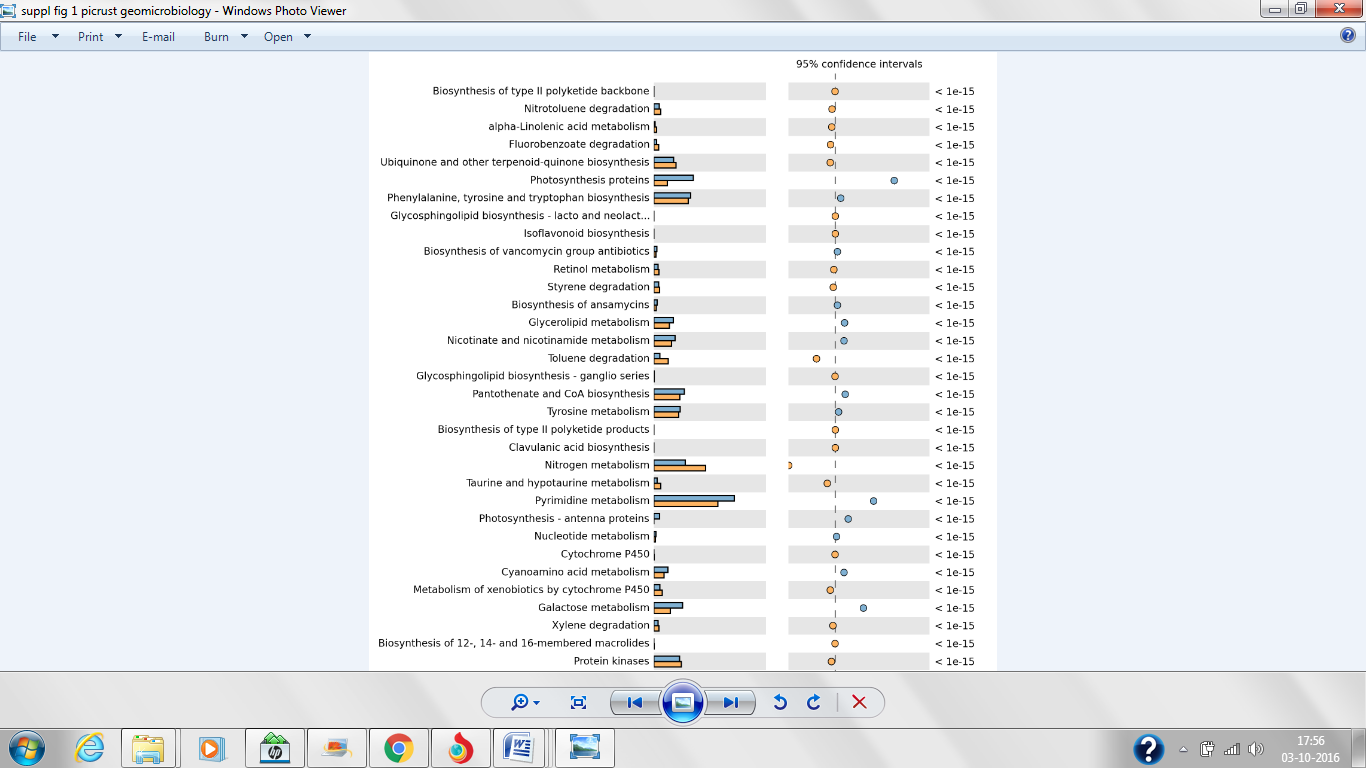


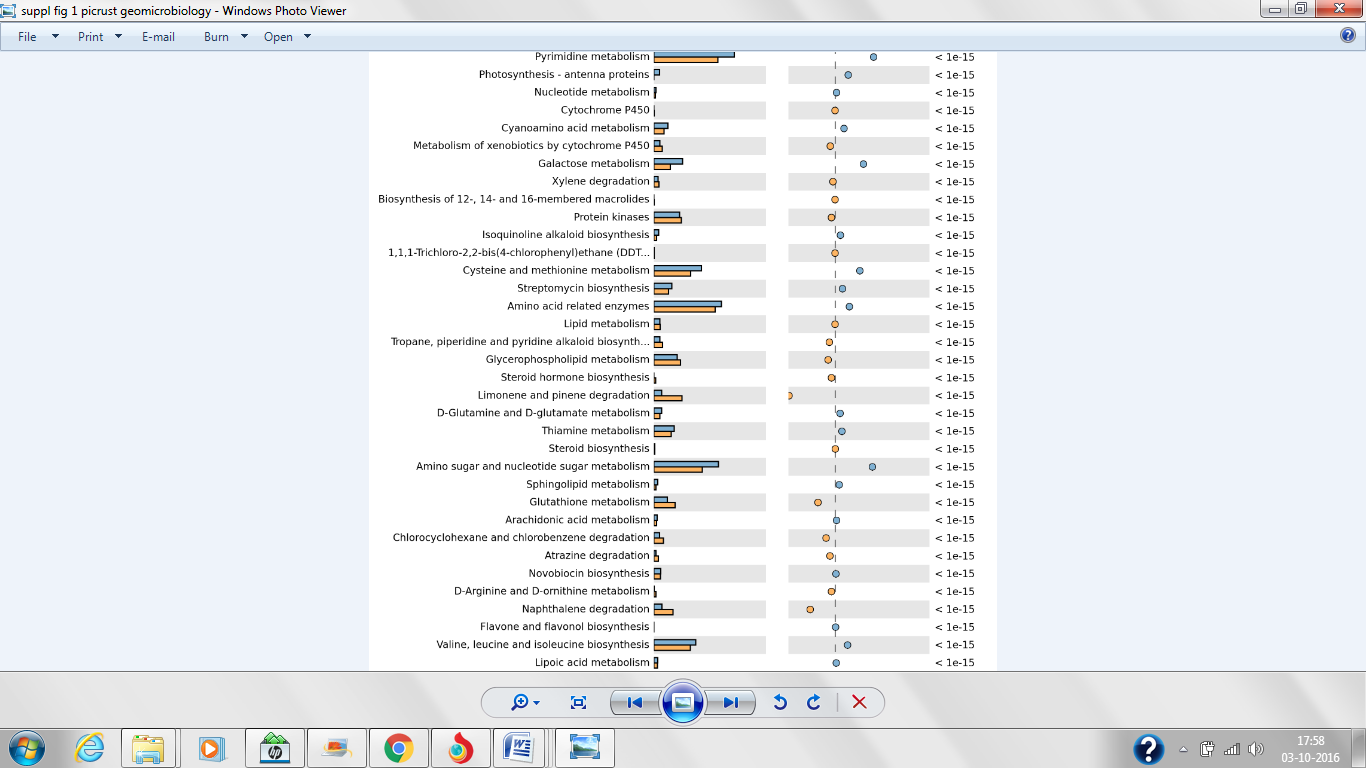


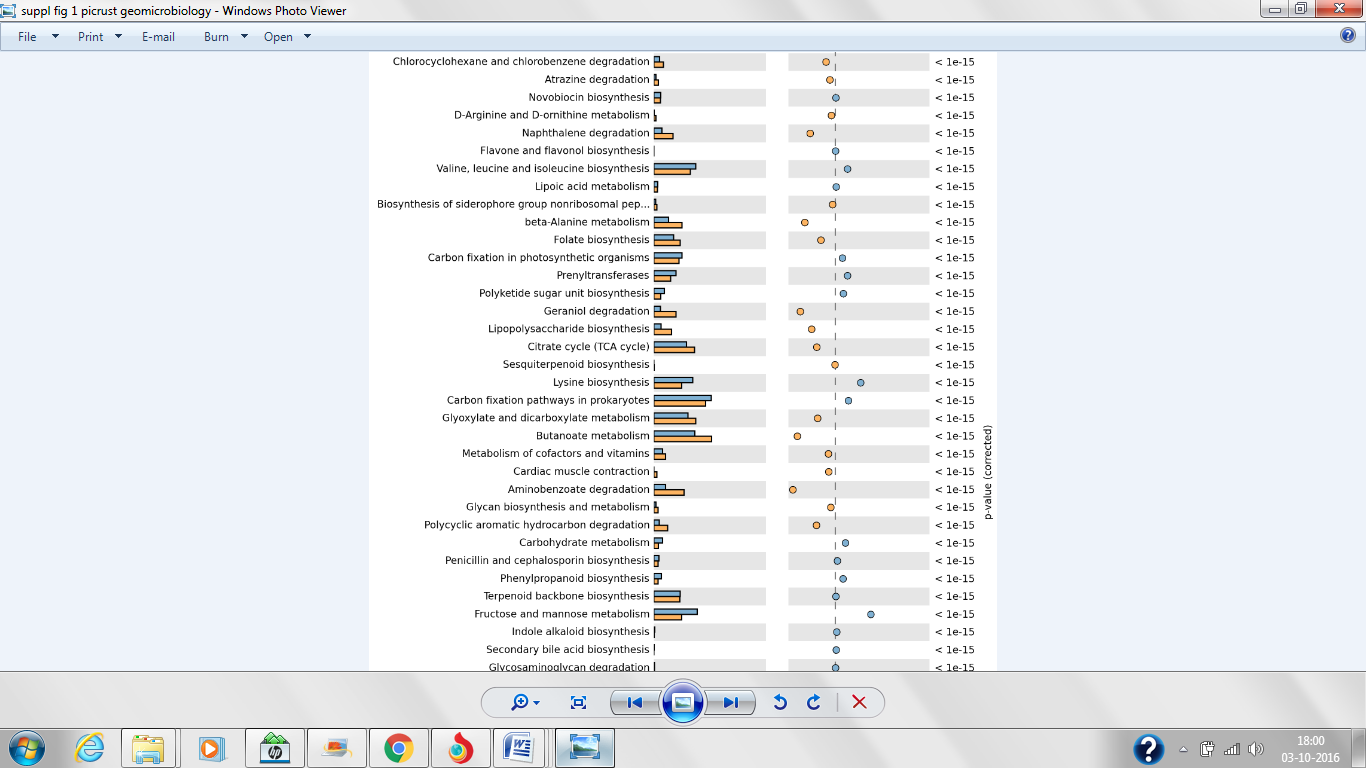


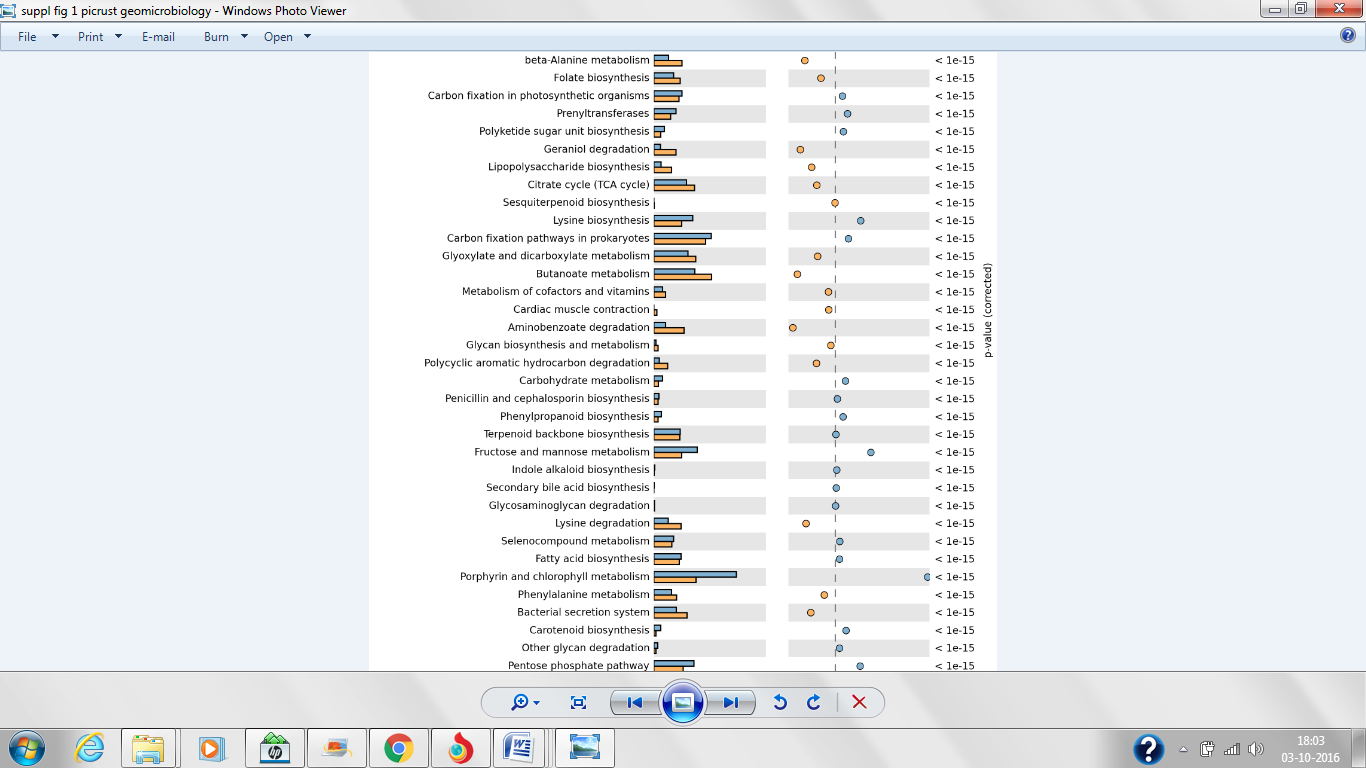


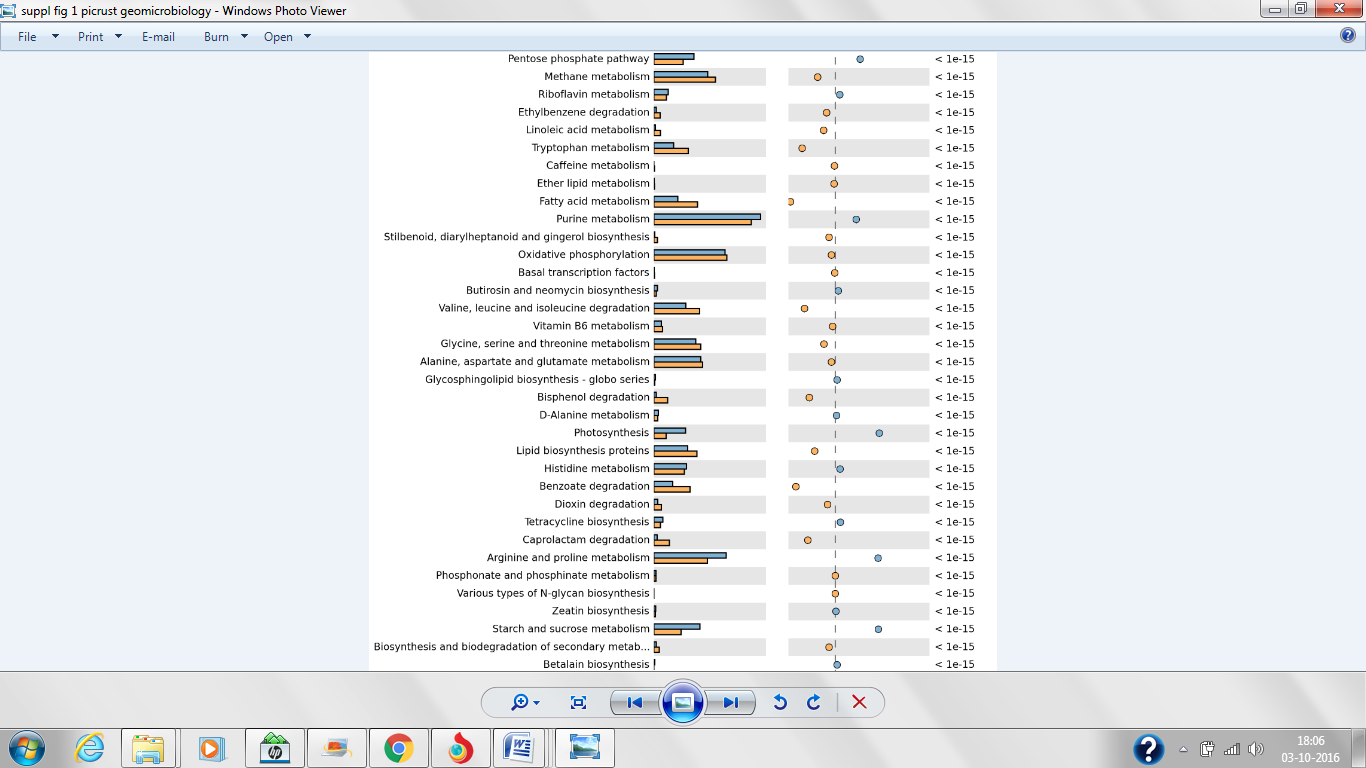


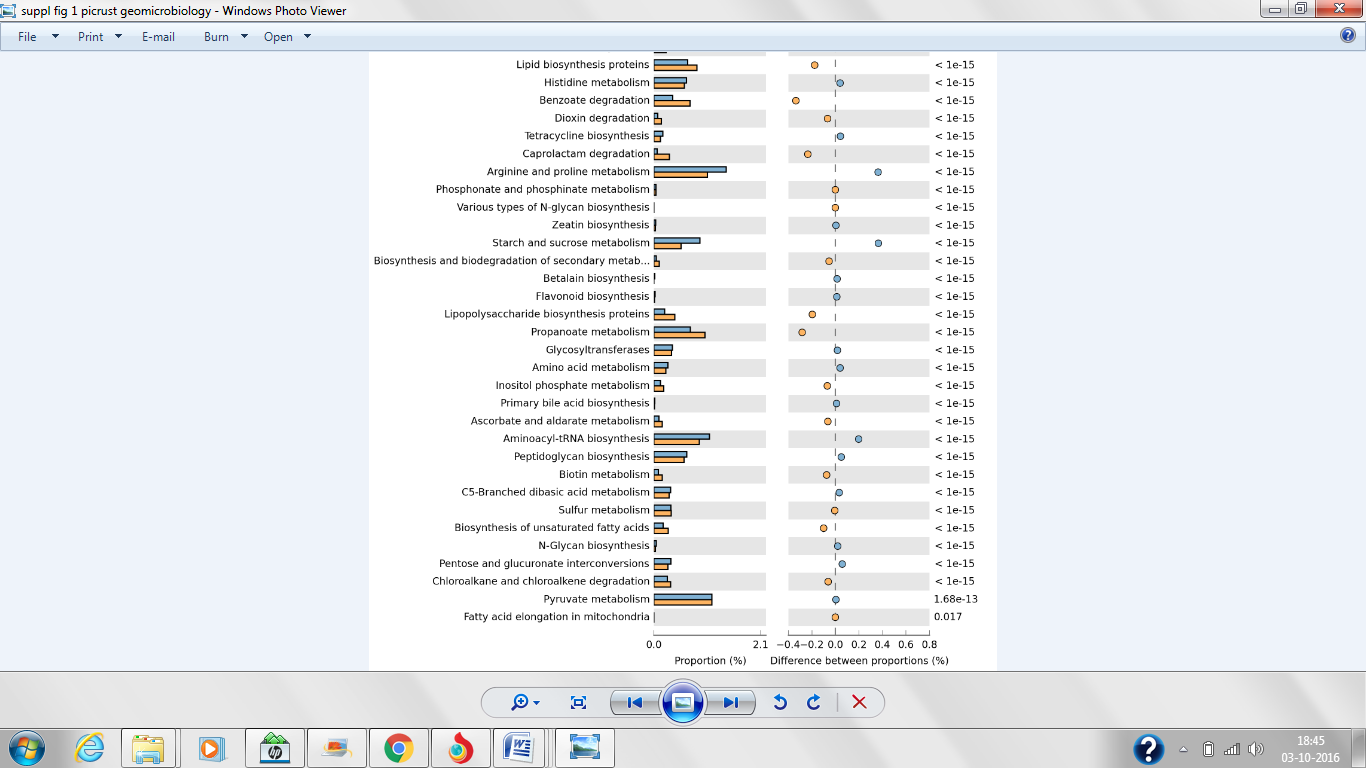


Fig. S1. **Relative abundance of genes in the two hot spring samples (orange color: Yumthang hot spring, blue color: Jakrem hot spring) for selected functional KEGG pathways inferred from 16S rRNA gene data using PICRUSt**
